# Central Role of Dendritic Cells in Pulmonary Arterial Hypertension in Human and Mice

**DOI:** 10.3390/ijms22041756

**Published:** 2021-02-10

**Authors:** Denise van Uden, Thomas Koudstaal, Jennifer A. C. van Hulst, Ingrid M. Bergen, Chelsea Gootjes, Nicholas W. Morrell, Geert van Loo, Jan H. von der Thüsen, Thierry P. P. van den Bosch, Maria-Rosa Ghigna, Frédéric Perros, David Montani, Mirjam Kool, Karin A. Boomars, Rudi W. Hendriks

**Affiliations:** 1 Department of Pulmonary Medicine, Erasmus MC, University Medical Center Rotterdam, 3015 GD Rotterdam, The Netherlands; d.vanuden@erasmusmc.nl (D.v.U.); t.koudstaal.1@erasmusmc.nl (T.K.); j.vanhulst@erasmusmc.nl (J.A.C.v.H.); i.bergen@erasmusmc.nl (I.M.B.); chelsea.gootjes@hotmail.com (C.G.); m.kool@erasmusmc.nl (M.K.); 2 Department of Medicine, University of Cambridge & NIHR BioResource for Translational Research & Addenbrooke’s Hospital NHS Foundation Trust & Royal Papworth Hospital NHS Foundation Trust, Cambridge CB2 0QQ, UK; nwm23@cam.ac.uk; 3 VIB Center for Inflammation Research, 9052 Ghent, Belgium; geert.vanloo@irc.vib-ugent.be; 4 Department of Biomedical Molecular Biology, Ghent University, 9052 Ghent, Belgium; 5 Department of Pathology, Erasmus MC, University Medical Center Rotterdam, 3015 GE Rotterdam, The Netherlands; j.vonderthusen@erasmusmc.nl (J.H.v.d.T.); t.vandenbosch@erasmusmc.nl (T.P.P.v.d.B.); 6 School of Medicine, Université Paris-Saclay, 94270 Le Kremlin-Bicêtre, France; mrghigna@gmail.com (M.-R.G.); frederic.perros@gmail.com (F.P.); davidmontani@gmail.com (D.M.); 7 INSERM UMR_S 999, Pulmonary Hypertension: Pathology and Novel Therapies, Hôpital Marie Lannelongue, 92350 Le Plessis Robinson, France; 8 Division of Pathology, Marie Lannelongue Hospital, 92350 Le Plessis Robinson, France; 9 Department of Respiratory and Intensive Care Medicine, Pulmonary Hypertension National Referral Center, Assistance Publique—Hôpitaux de Paris (AP-HP), Hôpital Bicêtre, 94270 Le Kremlin-Bicêtre, France

**Keywords:** pulmonary arterial hypertension, dendritic cells, inflammation, *Tnfaip3*, BMPR2, Toll-like receptor

## Abstract

The pathogenesis of idiopathic pulmonary arterial hypertension (IPAH) is not fully understood, but evidence is accumulating that immune dysfunction plays a significant role. We previously reported that 31-week-old *Tnfaip3^DNGR1-KO^* mice develop pulmonary hypertension (PH) symptoms. These mice harbor a targeted deletion of the TNFα-induced protein-3 (*Tnfaip3*) gene, encoding the NF-κB regulatory protein A20, specifically in type I conventional dendritic cells (cDC1s). Here, we studied the involvement of dendritic cells (DCs) in PH in more detail. We found various immune cells, including DCs, in the hearts of *Tnfaip3^DNGR1-KO^* mice, particularly in the right ventricle (RV). Secondly, in young *Tnfaip3^DNGR1-KO^* mice, innate immune activation through airway exposure to toll-like receptor ligands essentially did not result in elevated RV pressures, although we did observe significant RV hypertrophy. Thirdly, PH symptoms in *Tnfaip3^DNGR1-KO^* mice were not enhanced by concomitant mutation of bone morphogenetic protein receptor type 2 (*Bmpr2*), which is the most affected gene in PAH patients. Finally, in human IPAH lung tissue we found co-localization of DCs and CD8+ T cells, representing the main cell type activated by cDC1s. Taken together, these findings support a unique role of cDC1s in PAH pathogenesis, independent of general immune activation or a mutation in the *Bmpr2* gene.

## 1. Introduction

Pulmonary arterial hypertension (PAH) is characterized by structural remodeling of the arterial vasculature of the lung with a mean pulmonary arterial pressure (PAP) at rest of ≥20 mmHg, a mean capillary wedge pressure of ≤15 mmHg and a pulmonary vascular resistance of ≥3 Wood units [1]. This high pulmonary pressure causes hypertrophy of the right ventricle (RV), which can lead to heart failure and eventually death. Individuals with PAH are classified into World Health Organization (WHO) subgroups based on etiology and predisposing factors, such as genetic mutations classifying heritable PAH (HPAH) or systemic autoimmunity classifying connective tissue disease PAH (CTD-PAH). PAH patients with no known predisposing factors are classified as idiopathic PAH (IPAH) [1].

The major mutation found in HPAH patients is in the bone morphogenetic protein receptor type 2 (*BMPR2*) gene, causing loss of function or reduced receptor signaling (reviewed in [2]). BMPRII, which belongs to the transforming growth factor-β (TGFβ) receptor family, is expressed by various cell types, including vascular pulmonary endothelial cells and pulmonary artery smooth muscle cells (PASMCs), and is crucial in vascular homeostasis [3]. Reduced BMPRII signaling is associated with endothelial dysfunction and is not solely present in HPAH patients, but also in 14–35% of PAH patients—so-called sporadic cases without a known family history [4,5]. In the absence of a *BMPR2* gene mutation, reduced BMPRII signaling can be induced by numerous causes, including aberrant activity of the immune system (reviewed in [6]). Conversely, not all patients with a *BMPR2* mutation develop PAH, indicating that additional factors are needed to cause disease.

Several lines of evidence support the critical involvement of the immune system in PAH pathogenesis. Firstly, blood expression profiling and transcriptomic studies in patients and in a rat model showed an enrichment of toll-like receptor (TLR) signaling [7,8,9,10]. PASMCs and dendritic cells (DCs) of the immune system use these TLRs to sense pathogens [11,12]. Specific TLR activation on PASMCs leads to the production of chemokines, such as the X-X-C-motif chemokine ligand 8 (CXCL8 or interleukin (IL)-8) and CXCL10 (IP-10), and endothelin-1, which may participate in PAH pathogenesis [11]. Infections with viruses such as HIV, human herpes virus 8 and hepatitis B and C are associated with PAH development [13]. Host defense to bacteria are also thought to contribute to disease by autoantigen-related molecular mimicry [14]. Nevertheless, only a minor proportion of infected individuals develop PAH. Secondly, serum levels of particular pro-inflammatory cytokines, including IL-6, IL-8, IL-10 and IL-12p70, are elevated in PAH patients and are inversely correlated with survival [15]. Unsupervised analysis of blood proteomic profiles showed that PAH patients have distinct immune phenotypes comprising multiple cytokines, chemokines and growth factors, which are independent of WHO subgroups and correlate with clinical risk [15,16]. Thirdly, lung biopsies from IPAH patients often contain accumulations of lymphoid cells that form tertiary lymphoid organs (TLOs) [17], in which DCs are present and crucial for TLO maintenance [18,19].

DCs form the bridge between the innate and adaptive immune system. They take up antigens and, after processing, present these to T cells, thereby inducing an adaptive immune response. Several DC subsets exist under steady state conditions, including (i) plasmacytoid DC (pDC) which are known for their interferon production, (ii) type 1 conventional DCs (cDC1s) that excel in cross-presentation of antigens, and (iii) type 2 cDCs (cDC2s) which are known for CD4+ T cell induction [12]. Under inflammatory conditions, monocytes often differentiate to monocyte-derived-DCs (mo-DCs). Most of the DC subsets have been implicated in the pathobiology of IPAH and CTD-PAH (reviewed in [20]). CD209+ DCs are also massively recruited in remodeled arteries of IPAH patients and OX-62+ DCs accumulate with the same pattern in different rat models of severe pulmonary hypertension (PH), induced by monocrotaline or exposure to SU5416 and hypoxia [21,22,23]. Importantly, not only is the abundance of DCs different between PAH patients and healthy individuals, but also their activation status [24,25,26]. Further support for the involvement of DCs comes from the *Tnfaip3^DNGR1-KO^* mouse model, in which the TNFα-induced protein-3 (*Tnfaip3*) gene encoding the A20 protein, a negative regulator of the nuclear factor kappa B (NF-kB) signaling pathway, is deleted in DCs using the Cre-LoxP system [27]. In this model, the *Tnfaip3* gene is mainly targeted in cDC1s, whereby Cre expression is driven by the Dngr1 promoter. *Tnfaip3^DNGR1-KO^* mice spontaneously develop PH over time and show lymphocytic infiltration and vascular remodeling [27].

The exact mechanism by which *Tnfaip3^DNGR1-KO^* mice develop PH symptoms and the importance of the altered activation status of DCs or other immune cells in this model is not known. In our previous study we closely investigated the PH phenotype and the immunological landscape of the lungs of *Tnfaip3^DNGR1-KO^* mice. In this study we first characterized the immunological landscape of the heart in *Tnfaip3^DNGR1-KO^* mice. Moreover, since signs of PH are absent in young *Tnfaip3^DNGR1-KO^* mice, we wondered whether additional triggers might promote earlier development of the PH phenotype. Therefore, we investigated the effects of immune activation on PH development and lung inflammation by airway exposure to TLR ligands in vivo. By crossing *Tnfaip3^DNGR1-KO^* mice with *Bmpr2^+/R899X^* mice, we determined whether a vascular trigger would result in enhancement of the PH phenotype. Finally, we explored the relevance of our findings by determining DC and CD8+ T cell co-localization in human IPAH lung tissue.

## 2. Results

### 2.1. Increased Myocardial Infiltration of Dendritic Cells in the RV of Tnfaip3^DNGR1-KO^ Mice

**Figure 1:**
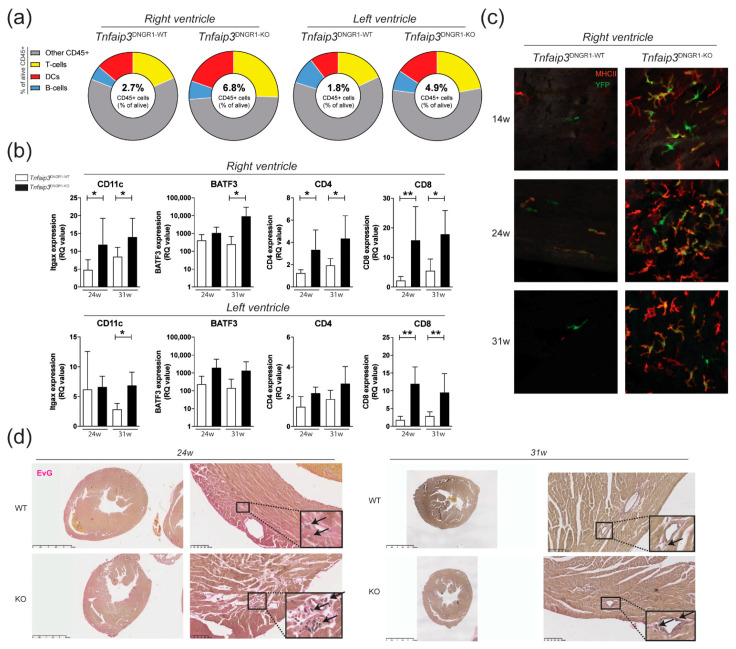
Increased myocardial infiltration of dendritic cells (DCs) in the right ventricle (RV) of *Tnfaip3^DNGR1-KO^* mice. (**a**) Flow cytometry analysis for CD45+ cells (alive CD45^+^ cells), DCs (CD3-/CD19-, MHC-II+CD11c+), T cells (CD3+) and B cells (CD19+) in separately measured right and left ventricle cell suspensions from hearts of *Tnfaip3^DNGR1-WT/KO^* mice; (**b**) mRNA expression (relative to the glyceraldehyde-3-phosphate dehydrogenase (GAPDH) housekeeping gene) of DC markers CD11c and BATF3, and the T cell subset markers CD4 and CD8 in 24- and 31-week-old *Tnfaip3^DNGR1-KO^* and wild-type (WT) control mice; (**c**) whole mount analysis of right ventricle staining for YFP (green) and MHCII (red) in age 14-, 24- or 31-week-old *Tnfaip3^DNGR1-KO^* mice. Results are presented as mean values + standard deviations of 4–6 mice per group. * *p* < 0.05, ** *p* < 0.01; (**d**) Elastin van Gieson (EvG)-stained whole heart section histology of *Tnfaip3^DNGR1-WT^* and *Tnfaip3^DNGR1-KO^* mice for representative sections. Scale in left panels is 2.5 mm and in right panels 100 µm. Arrows indicate lymphocytes.

*Tnfaip3^DNGR1-KO^* mice show increased RV pressures, RV hypertrophy and increased numbers of pulmonary DCs [27]. Flow cytometric analysis showed an increase in the proportions of DCs in the RV and of T cells in both the RV and left ventricle (LV) of *Tnfaip3^DNGR1-KO^* mice, compared with wild-type (WT) control mice at 31 weeks of age (Figure 1a, Supplementary Figure S1). The increase in the proportions of DCs and T cells was supported by RT-PCR analysis for the expression of the DC markers CD11c and basic leucine zipper transcriptional factor ATF-like 3 (BATF3), and the T cell subset markers CD4 and CD8, respectively (Figure 1b). To confirm the presence of DCs in the RV, we performed histological analysis on the RV of *Tnfaip3^DNGR1-KO^* and WT mice. Strikingly, in contrast to WT control mice, we found high number of DCs that were YFP^+^, indicating *Tnfaip3* gene deletion in the RV of *Tnfaip3^DNGR1-KO^* mice from 14 weeks of age onwards (Figure 1c). An Elastin van Gieson (EvG) staining for collagen and vascular intima wall thickening in heart tissue showed signs of lymphocytic infiltration and RV hypertrophy in 24-week-old *Tnfaip3^DNGR1-KO^* mice without evidence for myocardial vascular remodeling, when compared to WT mice (Figure 1d).

Thus, in *Tnfaip3^DNGR1-KO^* mice, lymphocyte infiltration was observed in both ventricles and myocardial DC infiltration in the RV was present from 14 weeks onwards.

### 2.2. TLR4 Activation Leads to RV Hypertrophy but Not to Increased RV Pressures in Young Tnfaip3^DNGR1-KO^ Mice

**Figure 2:**
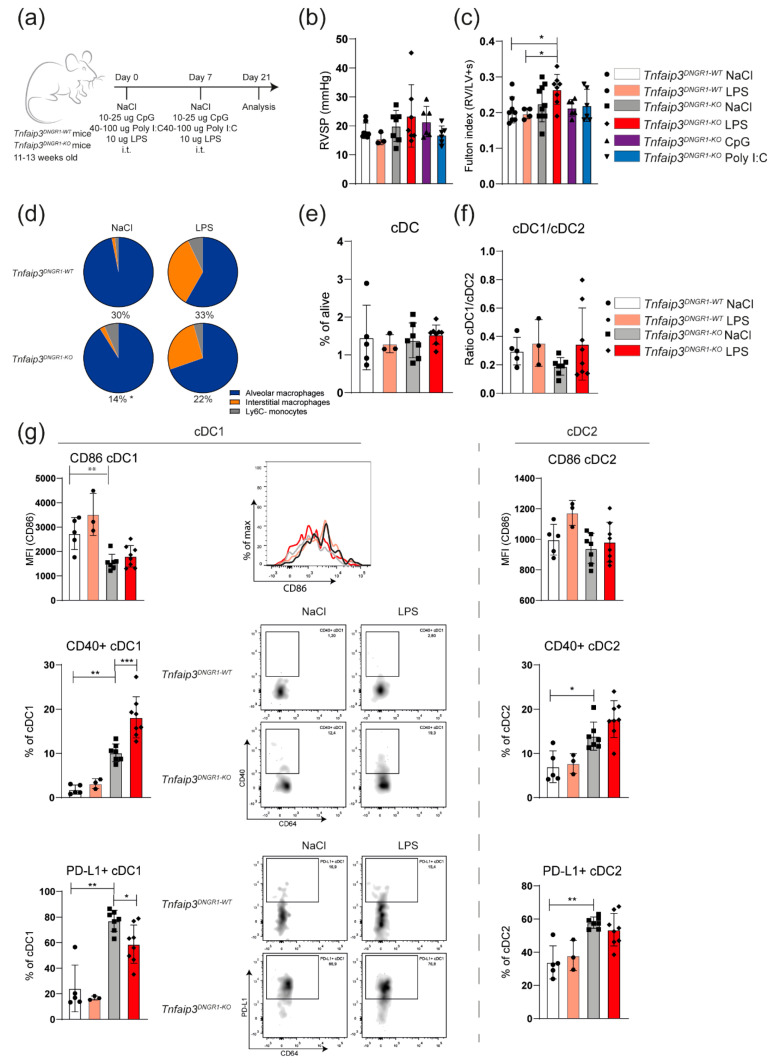
TLR4 activation leads to RV hypertrophy and cDC1 phenotype changes in *Tnfaip3^DNGR1-KO^* mice. (**a**) Scheme of intratracheal (i.t.) administration of CpG, lipopolysaccharide (LPS) and polyinosinic-polycytidylic acid (Poly I:C) in WT and *Tnfaip3^DNGR1-KO^* mice on day 0 and 7, and analysis on day 21; (**b**) right ventricular systolic pressure (RVSP) in toll-like receptor (TLR)-exposed WT and *Tnfaip3^DNGR1-KO^* mice, determined by right heart catheterization; (**c**) hypertrophy of RV measured by Fulton index (right ventricle/ left ventricle + septum); (**d**) assessment of the indicated cell types in LPS-exposed WT and *Tnfaip3^DNGR1-KO^* mice. Proportion of the CD64^+^GR1^−^ macrophage/monocyte population from total alive cells is indicated below the pie charts; (**e**,**f**) quantification of total DCs (**e**) and the CD103+ cDC1/CD11b+ cDC2 subset ratio (**f**) in the lungs of the indicated mice, as determined by flow cytometry; (**g**) quantification of surface CD86, CD40 and PD-L1 expression on CD103+ cDC1s (left) and CD11b+ cDC2s (right) in the indicated mouse groups. Flow cytometry analyses are shown as histogram overlays of CD86 expression (top), as dot plots with CD64/CD40 profiles (middle) and dot plots with CD64/PD-L1 profiles (bottom) of gated cDC1s. Results are presented as mean values + standard deviation of 3–10 mice (**b**,**c**) or 3–6 (**e**–**g**) mice per group. MFI = median fluorescence intensity. * *p* < 0.05, ** *p* < 0.01, *** *p* < 0.001.

The first signs of immune cell involvement in the *Tnfaip3^DNGR1-KO^* mice were already present at 14 weeks of age when the right ventricular systolic pressure (RVSP) was not yet elevated (Figure 1a). Since bacteria and viruses may be inducers of PAH [13,14], we wondered if an additional inflammatory trigger, such as exposure to TLR ligands, would result in development of PH at younger age. Therefore, 11–13-week-old *Tnfaip3^DNGR1-KO^* were intratracheally (i.t.) challenged with TLR4 ligand lipopolysaccharide (LPS), TLR3 ligand polyinosinic-polycytidylic acid (Poly I:C) or TLR9 ligand CpG, which was repeated after 7 days (Figure 2a). Two weeks after the last exposure, mice were analyzed for RV pressures, RV hypertrophy and immune cell involvement. In these experiments, LPS-treated or saline (NaCl)-treated WT mice were included as controls.

Administration of LPS or Poly I:C, but not CpG, induced weight loss in *Tnfaip3^DNGR1-KO^* mice (Supplementary Figure S2). This was also seen following LPS administration in WT mice. Exposure of *Tnfaip3^DNGR1-KO^* mice to TLR ligands did not result in increased RV pressures, compared to saline-treated *Tnfaip3^DNGR1-KO^* or WT mice that were administered saline or LPS (Figure 2b). However, in LPS-exposed *Tnfaip3^DNGR1-KO^* mice, the RV was larger than in *Tnfaip3^DNGR1-KO^* mice treated with saline, Poly I:C or CpG, or in LPS- or saline-treated WT mice (Figure 2c). As shown by EvG staining (Supplementary Figure S3), pulmonary vessels of LPS-exposed *Tnfaip3^DNGR1-KO^* and WT mice appeared to be thickened compared to saline-treated WT mice. Moreover, LPS exposure in *Tnfaip3^DNGR1-KO^* mice led to inflammatory infiltrates around the vessels. Thus, LPS exposure alone was not sufficient to cause elevation of the Fulton index, but in combination with the altered immune system in *Tnfaip3^DNGR1-KO^* mice, this was sufficient.

In these analyses, we found that the total population of monocytes/macrophages, which have also been implicated in PH pathogenesis [28], was decreased in saline- or LPS-exposed *Tnfaip3^DNGR1-KO^* mice (14 and 22 percent, respectively) compared to WT control mice (30 and 33 percent, respectively) (Figure 2d, see for gating: Supplementary Figure S4a). The induction of immune activation by LPS in *Tnfaip3^DNGR1-KO^* and WT mice was confirmed by the observation of increased proportions of interstitial macrophages (IM) in the lung [29] (Figure 2d).

The frequency of pulmonary cDCs or the cDC1/cDC2 ratio in *Tnfaip3^DNGR1-KO^* and WT mice did not significantly change after LPS exposure (Figure 2e,f; see for gating: Supplementary Figure S4b). Consistent with our reported findings in 31-week-old mice [27], we found altered expression of the cell surface markers CD86, CD40 and PD-L1 on cDCs in 14–17-week-old *Tnfaip3^DNGR1-KO^* mice, compared to WT mice (Figure 2g). LPS exposure increased CD40 and reduced PD-L1 expression but did not affect CD86 expression on cDC1s in the lungs of *Tnfaip3^DNGR1-KO^* mice. These changes in activation marker expression 2 weeks following LPS exposure were not seen in cDC2s in *Tnfaip3^DNGR1-KO^* mice, nor in either subset in WT mice (Figure 2g). 

In summary, TLR stimulation—mimicking bacterial or viral infection—did not lead to increased RV pressures and therefore did not appear to promote a PH phenotype in young *Tnfaip3^DNGR1-KO^* mice. However, LPS exposure in *Tnfaip3^DNGR1-KO^* mice was associated with RV hypertrophy and an altered surface phenotype of cDC1s but not cDC2s in the lung.

### 2.3. The PH Phenotype of Tnfaip3^DNGR1-KO^ Mice Is Not Enhanced by Concomitant BMPR2 Mutation

Next, we wondered if involving structural cells would result in the enhancement of the PH phenotype in *Tnfaip3^DNGR1-KO^* mice. To this end, we employed *Bmpr2^+/R899X^* mice, harboring a heterozygous knock-in allele of the R899X mutation as is seen in HPAH patients, which results in a premature stop codon [30,31]. These mice develop spontaneous PH by the age of 6 months, likely due to defective BMPRII expression in pulmonary endothelial cells [31].

**Figure 3:**
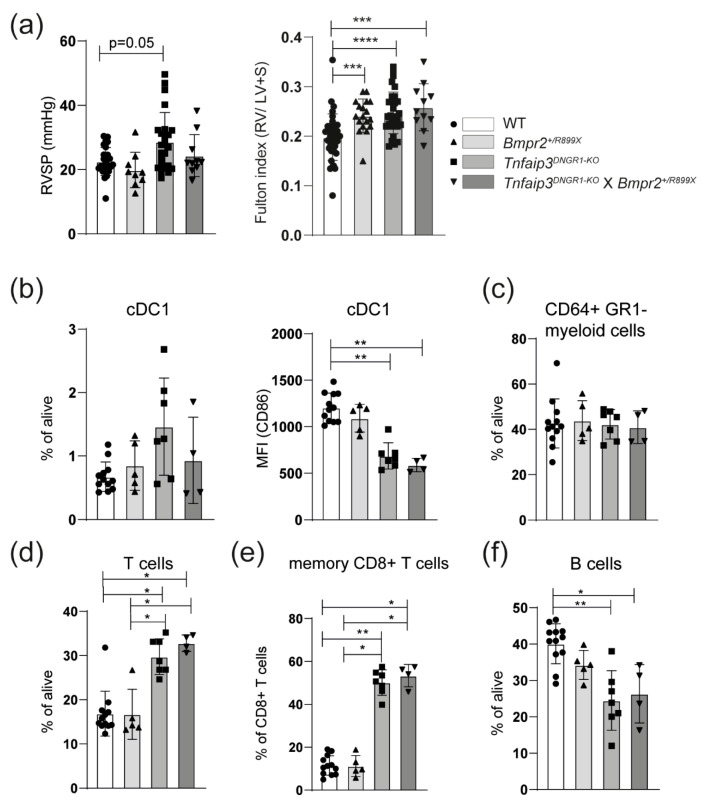
Concomitant *Bmpr2* mutation does not alter the pulmonary hypertension (PH) phenotype in *Tnfaip3^DNGR1-KO^* mice. (**a**) Right ventricular systolic pressure (RVSP), determined by right heart catheterization and hypertrophy of RV measured by Fulton index (right ventricle/ left ventricle + septum) of the indicated mouse groups; (**b**) quantification of lung CD103+CD11c+ cDC1 frequencies and CD86 expression (MFI = median fluorescence intensity) by flow cytometry; (**c**–**f**) quantification of CD64+GR1- monocyte/macrophage population (**c**), CD3+ T cells (**d**), memory CD44+CD62L- CD8+ T cells (**e**) or CD19+ B cells (**f**) determined by flow cytometry in the lungs of the indicated mouse groups. Results are presented as mean + standard deviation of 10–31 mice (**a**) or 4–12 mice (**b**–**e**) per group. * *p* < 0.05, ** *p* < 0.01, *** *p* < 0.001, **** *p* < 0.0001.

The *Tnfaip3^DNGR1-KO^* mice were crossed with *Bmpr2^+/R899X^* mice and analyzed at 31 weeks. Compared to WT mice, RV pressure was significantly increased in *Tnfaip3^DNGR1-KO^* mice, but not in *Bmpr2^+/R899X^* or *Tnfaip3^DNGR1-KO^* X *Bmpr2^+/R899X^* mice (Figure 3a). In all three mutant mouse groups the Fulton index was significantly higher than in WT mice (Figure 3a). A hematoxylin and eosin (H&E) staining showed that both *Tnfaip3^DNGR1-KO^* and *Tnfaip3^DNGR1-KO^* X *Bmpr2^+/R899X^* mice had signs of inflammatory infiltrates, which were not seen in WT and *Bmpr2^+/R899X^* mice (Supplementary Figure S5). Thickening of the small and mid-sized pulmonary vessels was evident in all three mutant mouse groups (shown by EvG and α smooth muscle actin (α-SMA) staining in Supplementary Figure S5). However, the RV pressures were only increased in *Tnfaip3^DNGR1-KO^* mice (Figure 3a).

We subsequently investigated whether the *Bmpr2^+/R899X^* genotype affected immune cells in the lung. The frequency of the cDC1 subset was similar across the different mouse groups (Figure 3b). Surface CD86 expression on cDC1s was specifically reduced in *Tnfaip3^DNGR1-KO^* mice, irrespective of the *Bmpr2* genotype (Figure 3b). In all three mutant mouse groups, the CD64+GR1- macrophage/monocyte populations were unaltered (Figure 3c). The proportions of T cells in the lung were significantly increased in *Tnfaip3^DNGR1-KO^* mice, irrespective of the *Bmpr2* genotype (Figure 3d). Hereby, the proportions of CD4+ and CD8+ T cells were similar in the four mouse groups (Supplementary Figure S6). The proportions of CD44+CD62L- memory cells within the CD8+ T cell population were increased in *Tnfaip3^DNGR1-KO^* mice, irrespective of the *Bmpr2* genotype (Figure 3e). Finally, the frequency of pulmonary B cells was slightly lower in *Tnfaip3^DNGR1-KO^* mice, irrespective of the *Bmpr2* genotype, compared to WT or *Bmpr2^+/R899X^* mice (Figure 3f).

Overall, *Tnfaip3^DNGR1-KO^* X *Bmpr2^+/R899X^* mice did not show increased RV pressure and immunologically paralleled the *Tnfaip3^DNGR1-KO^* mice. From these findings we conclude that *Tnfaip3^DNGR1-KO^* cDC1 cells have a unique and dominant ability to induce PH symptoms, which is not modulated or enhanced by aberrant pulmonary endothelial cells in *Bmpr2^+/R899X^* mice.

### 2.4. DCs and CD8+ T Cells Co-Localize around Blood Vessels in Lungs of IPAH Patients

The cDC1 subset excels in cross presentation and is known for its ability to activate CD8+ T cells [12]. Since cDC1s seem to have a unique and dominant ability to induce a PH phenotype, we determined the presence of DCs and CD8+ T cells in both *Tnfaip3^DNGR1-KO^* mice and human IPAH lung tissue by immunohistochemistry.

**Figure 4:**
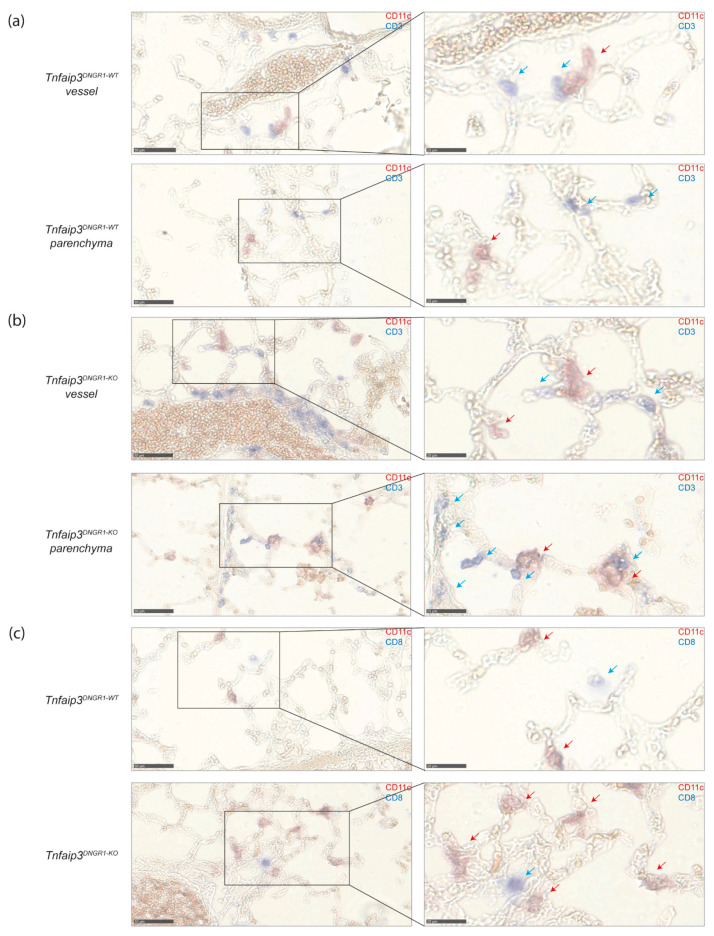
DCs and T cells are more often in close proximity around vessels and in parenchyma in *Tnfaip3^DNGR1-KO^* mice. (**a**) DC (CD11c, red) and T cell (CD3, blue) staining on lung cryosections of 31-week-old WT mice of which an area around a vessel (top) and parenchyma (bottom) of the same mouse is depicted. Magnification of selected areas (right); (**b**) DC (CD11c, red) and T cell (CD3, blue) staining on lung cryosections of 31-week-old *Tnfaip3^DNGR1-KO^* mice of which an area around a vessel (top) and parenchyma (bottom) of the same mouse is depicted. Magnification of selected areas (right); (**c**) DC (CD11c, red) and CD8+ T cells (CD8, blue) of WT (top) and *Tnfaip3^DNGR1-KO^* (bottom) mice around a vessel. Magnification of selected area (right). Data shown are representative for 4–6 mice per group. Scale in left panels is 50 µm and of right panels 25 µm. Blue arrows indicate T-cells, and red arrows indicate DCs in the larger magnification (right panels).

In the lungs of 31-week-old WT and *Tnfaip3^DNGR1-KO^* mice, DCs and T cells were identified by CD11c and CD3 expression, respectively (Figure 4a,b). Although some of these cells were observed in the lung parenchyma, most of the DCs and T cells were located in the vicinity of blood vessels, and their numbers were increased in *Tnfaip3^DNGR1-KO^* mice, as compared to WT mice (Figure 4a,b). In the lungs of *Tnfaip3^DNGR1-KO^* mice, DCs often were in close proximity to CD3+ T cells. A fraction of these T cells were CD8+ T cells, as shown by the staining for CD8 (Figure 4c).

**Figure 5:**
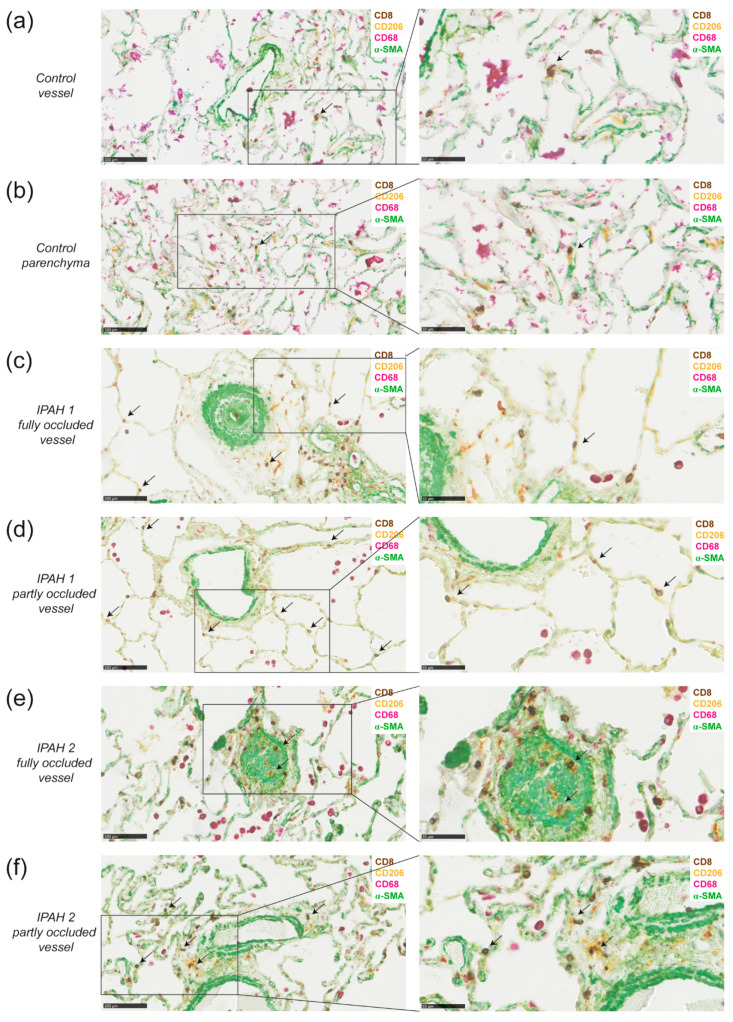
DCs are in close proximity to CD8+ T cells around vessels and in parenchyma in lungs of idiopathic pulmonary arterial hypertension (IPAH) patients. (**a**,**b**) 4-plex chromogenic multiplex staining of DCs (CD206+, yellow), macrophages (CD68+, purple with or without CD206), T cells (CD8, DAB) and α-SMA (green) around a vessel and intraparenchymal (**a**) or in parenchyma (**b**) and the magnification of the indicated area (right) in the same healthy tissue from a smoking patient who was diagnosed with adenocarcinoma; (**c**,**d**) determination of DCs (CD206+CD68-), CD8+ T cells (CD8+), macrophages (CD68+) and vessels (α-SMA) around a fully occluded vessel (**c**) or partly occluded vessel (**d**) and the magnification of the indicated area (right) of the same IPAH patient with moderate remodeling; (**e**,**f**) determination of DCs, CD8+ T cells, macrophages and vessels around a fully occluded vessel (**e**) or partly occluded vessel (**f**) and the magnification of the indicated area (right) of the same a IPAH patient with extensive remodeling and immune cell infiltration. Data shown are representative for 6 IPAH patients. Sporadic CD206+CD68- cells with a morphology suggesting neutrophil identity were not regarded as DCs. Scale in left panels is 100 µm and of right panels 50 µm. Arrows indicate DC and CD8 co-localization

To determine co-localization of DCs with CD8+ T cells in the lungs of IPAH patients, we studied the presence of these cells by chromogenic multiplex immunohistochemistry in paraffin-embedded lung tissue of six IPAH patients. DCs express several mannose receptors such as CD209 and CD206, therefore we used the mannose receptor CD206 to identify DCs [12]. Since macrophages also express CD206 we co-stained for CD68, which is only expressed by macrophages to distinguish DCs (CD68-CD206+) from macrophages (CD68+CD206+). The multiplex staining further included staining for CD8, and for α-SMA to identify blood vessel structures. In healthy lung tissue, the number of immune cells is generally limited, and immune cell infiltrates mostly consist of macrophages, while the numbers of DCs and CD8 cells are low (Figure 5a,b). Occasionally, we found CD8+ T cells close to CD68-CD206+ DCs (Shown for a control lung in Figure 5a,b).

In IPAH lungs, remodeling of pulmonary vessels was evident from the prominent staining with α-SMA (Figure 5c,e). In the lungs of all six IPAH patients analyzed, macrophages were present, and in two patients the numbers were high, forming dense macrophages infiltrates. DCs were abundantly detectable as CD68-CD206+ cells in lung tissue of all six IPAH patients, although their presence in the parenchyma and around arterioles varied between patients. Figure 5 shows two representative patients, one patient with limited remodeling (panel c, d) and one patient with more severe remodeling (panel e, f). Close proximity of DCs and CD8+ T cells, indicative for DC-CD8+ T cell interaction, was mostly observed near affected, partly occluded vessels, but less frequently near fully occluded vessels (Figure 5c–f, shown in higher magnification in the right panel). Macrophages (CD206+CD68+) were particularly present near fully occluded vessels. Notably, in IPAH lungs with extensive remodeling and immune cell infiltration, co-localization of DCs and CD8+ T cells was most prominent. IPAH patient 2 had more remodeling and showed more DC and CD8 interaction than IPAH patient 1, in which remodeling of the vessels was less severe.

In conclusion, these findings demonstrate close proximity of DCs and CD8+ T cells around vessels and in parenchyma in lungs of *Tnfaip3^DNGR1-KO^* mice and IPAH patients, supporting a role for cDC1s in PAH pathology.

## 3. Discussion

We have previously shown that targeted deletion of the *Tnfaip3* gene, encoding the NF-κB regulator A20, specifically in type I conventional dendritic cells (cDC1s), is sufficient to induce PAH symptoms in mice. In this study, we investigated the importance of the altered activation status of DCs in the development of PH.

We show that the heart, predominantly the RV, of *Tnfaip3^DNGR1-KO^* mice is infiltrated with MHCII+CD11c+ DCs, CD19+ B cells and CD3+ T cells. Exposing 11–13-week-old *Tnfaip3^DNGR1-KO^* mice to an additional TLR trigger did not lead to increased RV pressures and thereby a full PH phenotype. However, LPS exposure did induce hypertrophy of the RV and an altered activation of specifically cDC1s. We also found that adding a vascular trigger by reducing BMPRII expression in older *Tnfaip3^DNGR1-KO^* mice did not result in an enhanced PH phenotype. As in our *Tnfaip3^DNGR1-KO^* mouse model the Dngr1-cre-driven deletion mostly targets cDC1s, our findings indicate a key role of these cells in PAH development, which cannot be modulated by additional innate immune activation or defective BMPRII function in structural cells. cDC1s excel in cross presentation and thereby predominately interact with CD8+ T cells. DCs and T cells were mostly present near the small-to-mid-sized pulmonary vessels in *Tnfaip3^DNGR1-KO^* and WT mice. Importantly, this was confirmed by the presence of DCs in close proximity to CD8+ T cells mostly around affected, but not fully occluded, pulmonary vessels in the lungs of IPAH patients.

We demonstrate that immune cells are not only present in the lungs of *Tnfaip3^DNGR1-KO^* mice [27], but also in the heart, and especially in the RV. Using flow cytometry, we found higher frequencies of lymphocytes in the RV than in the LV of the hearts from both *Tnfaip3^DNGR1-KO^* and WT mice. Histological analysis of DCs in the RV showed more pronounced differences between WT and *Tnfaip3^DNGR1-KO^* mice than was seen by flow cytometry. An explanation might be that large local differences within RV regions exist between two mouse groups, which may be obscured in an analysis of whole RV cell suspensions, as is performed in flow cytometry. The RV is the ventricle that is enlarged in PAH and immune cells may be crucial in its remodeling and enlargement. However, it cannot be excluded that the influx of immune cells is a bystander effect, next to a primary process in the heart. Although the current knowledge on the presence of immune cells in the hearts of PAH patients is limited (reviewed in [32]), it has been shown that immune cells are increased in the hearts of SSc-PAH and IPAH patients compared to controls [33]. In SSc-PAH patients, immune cells were predominantly increased in the RV, but DCs were not specifically analyzed [33].

In our *Tnfaip3^DNGR1-KO^* mice, a large part of the cardiac CD45+ cells consisted of DCs. Moreover, we found that DCs were present in the RV before pressures in the RV were elevated. To our knowledge, this is the first study that shows increased presence of myocardial DCs in an inflammatory-driven murine model for PH. Further studies are necessary to investigate if this increased presence of myocardial DCs is also found in the heart of PAH patients.

A large proportion of the CD45+ hematopoietic cells appeared to be neither T cells, B cells nor DCs, but might be macrophages or monocytes, since these cells comprise a large population in the heart, both in health and disease (reviewed in [34,35]). Macrophages are implicated in PAH [25,28], possibly by inflammasome-dependent secretion of pro-inflammatory cytokines such as IL-1β and IL-18 [36,37], which are increased in serum of PAH patients [15]. Interestingly, it has been found in mice that A20-deficient macrophages show spontaneous inflammasome activity and IL-1β secretion [38,39]. It is therefore conceivable that in *Tnfaip3^DNGR1-KO^* mice, cDC1s also exhibit increased inflammasome activity and IL-1β secretion, and that A20-deficient cDC1s might therefore parallel pathogenic macrophages in PAH. However, it is also attractive to speculate that increased inflammasome-dependent IL-1β secretion stimulates potent cytotoxic T cell responses by CD8+ T cells, as was very recently reported in the context of anti-tumor immunity [40]. Further experiments are required to investigate the role of inflammasome activation in DC-T cell interaction in inflamed lymph nodes as well as in local myocardial inflammation in *Tnfaip3^DNGR1-KO^* mice.

Although 31-week-old *Tnfaip3^DNGR1-KO^* mice show signs of PH, these are absent in young *Tnfaip3^DNGR1-KO^* mice. We wondered whether additional triggers would lead to increased RV pressures in young *Tnfaip3^DNGR1-KO^* mice. It is believed that in order to develop PAH, a second trigger might be needed. For example, a viral infection alone is unlikely to cause PAH, since only one in 200 AIDS patients and only 10% of Hepatitis C patients show signs of PAH [13]. Therefore, we exposed 11–13-week-old *Tnfaip3^DNGR1-KO^* mice to TLR3, TLR4 and TLR9 triggers, but none of these led to increased RV pressures within 2 weeks after the last exposure. These findings indicate that the alterations in the A20-deficient DCs were already sufficient to induce increased pressures over time, and that RVSP was not substantially enhanced by the TLR triggers investigated. Although there was no significant increase in pressure, TLR4 triggering by LPS resulted in RV hypertrophy and had effects, even two weeks after the last triggering, on the activation phenotype of cDC1s in the *Tnfaip3^DNGR1-KO^* mice, but not in WT mice. The in vivo exposure to LPS increased CD40 expression and reduced PD-L1 expression on cDC1s in the lungs of *Tnfaip3^DNGR1-KO^* mice. In in vitro experiments, it has been demonstrated that activation of DCs by LPS can prime DCs for subsequent signals from T cells [41]. The observed increase in CD40 expression and decrease in PD-L1 expression on *Tnfaip3^DNGR1-KO^* cDC1s suggest a prolonged CD8+ T cell priming capacity of these cDC1s: at least up to two weeks after LPS exposure.

Reduced BMPRII signaling as an additional trigger in an inflammatory-based 31-week-old *Tnfaip3^DNGR1-KO^* mouse model did not result in an enhanced PH phenotype. This might be related to the mild PH phenotype seen in *Bmpr2^+/R899X^* mice [31]. *Bmpr2^+/R899X^* mice do show increased RV pressures at 6 months of age, but do not have an increased Fulton index, indicating that there is no RV hypertrophy. In our hands, however, the *Bmpr2^+/R899X^* mouse model did not display detectable increased RV pressures but did show remodeling of the pulmonary arteries and a higher Fulton index compared to WT controls. We did not find evidence for an additional effect of reduced BMPRII signaling on the immune system. The *Bmpr2^+/R899X^* mice showed no apparent changes in DCs, T cells or B cells. It was quite unexpected that there were no signs of altered immunity in the *Bmpr2^+/R899X^* mice, since it is known that BMPRII deficiency leads to the recruitment of lymphocytes, macrophages and neutrophils to the vessels [6]. Because there is a genotype-phenotype relationship regarding *Bmpr2* mutations [30], it might be that this is also the case for immune involvement. Therefore, we cannot exclude that a more severe BMPRII-deficient PH mouse model may display changes in the immune system and may enhance the PH symptoms in *Tnfaip3^DNGR1-KO^* mice.

In our immunohistochemical analyses, the lungs of *Tnfaip3^DNGR1-KO^* mice showed more CD3+ T cells and DCs than WT mice, mostly around vessels. These DCs and T cells might contribute to vessel remodeling, which is the major hallmark of PAH pathology. DCs are not only able to activate and attract pathogenic T cells, but also produce cytokines and chemokines such as IL-6 or CXCL13 [20]. Hereby, DCs may have a direct effect on the pulmonary vessels, since IL-6 may promote smooth muscle cell migration. Moreover, DCs may act indirectly by attracting other immune cells, for example B cells that may develop into plasma cells producing auto-antibodies recognizing endothelial epitopes. Likewise, attracted T cells may produce cytokines such as IL-17 that enhance fibroblast proliferation and collagen production [20]. In WT and *Tnfaip3^DNGR1-KO^* mice, only a minor fraction of the T cells in the lung appeared to be CD8+ T cells. This was in contrast to the lungs of IPAH patients, in which both CD8+ T cells and DCs were abundantly present, even though we noticed heterogeneity among the IPAH patients. It is known that DCs expressing mannose receptors (CD209) are increased in lung tissue of IPAH patients, compared to control tissues [25]. The presence of DCs around pulmonary vessels has been shown before [17], but it remained unknown whether at this location they would also interact with T cells. We found that in the lung tissue of IPAH patients, many DCs localized adjacent to CD8+ T cells. Interestingly, this co-localization was particularly observed near affected, not fully occluded vessels, but to a lesser extent near fully occluded and remodeled vessels. This might be explained by the role of DCs and CD8+ T cells in the process of inflammation and remodeling, which may be less present in fully occluded vessels [17]. Since cDC1s are known to have the capacity to interact and activate CD8+ T cells, the DCs adjacent to CD8+ T cells in the IPAH lungs are most likely cDC1s. However, additional markers such as IRF8 are needed to determine if these cells are indeed cDC1s.

To be able to explore synergistic effects on PH development in *Tnfaip3^DNGR1-KO^* mice, we chose a physiological model of BMPRII reduction, as this is a mild PH model that leaves a window for detection of an increase in disease severity. Although the *Bmpr2^+/R899X^* mice showed remodeling of the pulmonary vessels and an increase in Fulton index, we did not measure increased RV pressures. Therefore, it remains possible that we were unable to detect PH enhancement in *Tnfaip3^DNGR1-KO^* X *Bmpr2^+/R899X^* mice was due to absence of full PH symptoms in the *Bmpr2^+/R899X^* mice. On the other hand, it might be challenging to detect synergistic effects using a severe PH model, such as PH triggered by monocrotaline-induced endothelial damage or a more severe BMPRII-deficient PH mouse model [30,42].

Using the model of TLR4 triggering by LPS in the *Tnfaip3^DNGR1-KO^* mice, we observed vascular remodeling and RV hypertrophy, but not an increase in RVSP. However, we observed a trend of increased RVSP in *Tnfaip3^DNGR1-KO^* mice which did not reach significance. In this context, a limitation of our study is that it remains unclear whether multiple exposures of LPS, an analysis at later time points after LPS exposure, or exposure to TLR ligands other than LPS, CpG or Poly I:C would reveal more pronounced changes in RVSP in *Tnfaip3^DNGR1-KO^* mice.

Taken together, our findings in *Tnfaip3^DNGR1-KO^* mice suggest an important role of cDC1s in PAH pathogenesis, independent of general immune activation or a mutation in the *Bmpr2* gene. This unique role includes the engagement of CD8+ T cells, given the observed co-localization of DCs and CD8+ T cells around vessels in the lungs of aged *Tnfaip3^DNGR1-KO^* mice with PH symptoms and in the lungs of IPAH patients. Deeper knowledge about the functional role of DC subsets in the activation of T cells in PAH improves our understanding of PAH pathobiology and is expected to contribute to the identification of candidate therapeutical targets.

## 4. Materials and Methods

### 4.1. Mice

*Tnfaip3^DNGR1-KO^* mice (*Clec9a*^+/Cre^ X *Tnfaip3^fl/fl^*) have been described previously [27,43]. *Tnfaip3^DNGR1-WT^* mice (*Clec9a*^+/non-cre^ X *Tnfaip3^fl/fl^*) littermates served as WT controls. Mice were sacrificed and analyzed at 14–16 weeks. *Tnfaip3^DNGR1-KO^* mice were crossed with *Bmpr2^+/R899X^* mice, which have a premature stop codon in the *Bmpr2* gene leading to reduced BMPRII expression [31]. In the experiments, four groups of mice were analyzed: WT, single *Bmpr2^+/R899X^* mice, single *Tnfaip3^DNGR1-KO^* and *Bmpr2^+/R899X^* X *Tnfaip3^DNGR1-KO^* mice. Mice were sacrificed and analyzed when 31 weeks old. Mice were bred and housed at the Erasmus MC under SPF conditions. Experiments were approved by the animal ethical committee of the Erasmus MC, Rotterdam, The Netherlands.

### 4.2. Exposure of TLR Ligand in Tnfaip3^DNGR1-KO^ Mice

For exposure to TLR ligands, 11–13-week-old *Tnfaip3^DNGR1-KO^* and *Tnfaip3^DNGR1-WT^* mice were anesthetized using 2.5% isoflurane and subsequently administered intratracheally (i.t.) either 10–25 µg CpG (ODN 1668, Invitrogen, Waltham, MA, USA), 40–100 µg Poly I:C (Invivogen, San Diego, CA, USA), 10 µg LPS (Sigma-Aldrich, St. Louis, MO, USA) or 0.9% sodium chloride (NaCl) at day 0 and day 7. Mice were weighed daily and sacrificed at day 14 for analysis.

### 4.3. Right Heart Catheterization and Fulton Index

To determine RV pressures, mice were weighed and anesthetized by an intraperitoneal (i.p.) injection with urethane (2 mg/g, given according to weight of the mice). Urethane was used since this anesthetic has a minimal effect on the cardiovascular and respiratory system. After placing a tracheal canula (miniVent type 845, Hugo Sachs Elektronik, March-Hugstetten, Germany) the RV pressure was measured using a pressure catheter (Miller Inc., Houston, TX, USA), as described previously [27]. WinDaq (DataQ Instruments inc., Akron, OH, USA) and Matlab (the Mathworks inc., Natick, MA, USA) were used to record and analyze pressures. After excision of the heart, RV and left ventricle (LV) + septum (S) were separated and weighted separately [27]. The Fulton index was calculated by dividing the weight of the RV by the weight of LV+S.

### 4.4. RNA Extraction Real-Time Quantitative RT-PCR

Heart tissue (after separation of RV and LV/S) of *Tnfaip3^DNGR1-WT/KO^* mice was stored in −80 °C until processing of the material. Heart tissue was homogenized using lysis buffer with 2-mercaptoethanol and ¼’’ ceramic spheres (MP biomedicals, 6540-034) by shaking for 40 s (MP biomedicals, Fastprep-24 5G). Subsequently, RNA was extracted using TRI reagent (Sigma-Aldrich, T9424) and cDNA synthesis was performed using a RevertAid H minus First Strand cDNA synthesis Kit (Thermo Fisher Scientific, K1632, Waltham, MA, USA). The cDNA was used to measure expression of CD11c, BATF3, CD4 and CD8, as well as the Glyceraldehyde-3-phosphate dehydrogenase (GAPDH) housekeeping gene by RT-PCR in a 7300 Real time PCR system (Applied Biosystems, Foster City, CA, USA). Primer sequences are given in Supplementary Table S1.

### 4.5. Histology of Lung and Heart Tissues

Five-µm-thick paraffin-embedded mouse lung sections were stained with hematoxylin and eosin (H&E). Sections were stained for alpha smooth muscle actin (α-SMA) using mouse anti-human α-SMA-PE (R&D Systems, Minneapolis, MN, USA) and goat anti-PE-alkaline phosphatase (Rockland, Limerick, PA, USA) as primary and secondary antibody, respectively. Antibodies used for immunohistochemical staining are listed in Supplementary Table S2. Prior to α-SMA staining, antigen retrieval was performed by incubating slides in citrate buffer (PH 6.0, Sigma-Aldrich C9999-1000MC) at 78 ℃ for 10 min. Elastin van Gieson (EvG) staining (Elastic Stain kit (HT25), Sigma-Aldrich) was performed, according to manufacturer’s instructions. To determine co-localization of CD11c with either CD3+ or CD8+ T cells, lung slides were first stained with hamster anti-mouse CD11c PE (clone N418, Thermo Fisher Scientific) and rat anti-mouse CD3 APC-ef780 (clone 17A2, Thermo Fisher Scientific), or rat anti-mouse CD8 Fitc (clone 53-6.7, BD Bioscience, San Jose, CA, USA). Next, slides were stained by secondary antibodies goat anti-PE-alkaline phosphatase (Rockland) and goat anti-rat PE (Sigma-Aldrich).

Mouse hearts were either directly separated after section and embedded for whole mount analyses of the RV or fixed in total with 4% paraformaldehyde (PFA) (Carl Roth, Karlsruge, Germany) before paraffin embedding. Five-μm-thick paraffin-embedded heart sections were stained with EVG. For the evaluation of DCs in the heart, whole mount RVs were analysed. Directly after separation of the left ventricle/septum, the RV was pinned and fixed overnight using fresh 4% PFA/PBS. The next day, the RVs were washed and incubated with the primary antibody specific for MHCII and GFP (antibody details in Supplementary Table S2). After 24 h, the RVs were washed and incubated with the secondary antibody. Finally, the RVs were embedded and analysed using a meta311 confocal microscope.

For immunohistochemical analysis of human lung tissue, we performed a 4-plex chromogenic multiplex by automated IHC using the Ventana Benchmark Discovery ULTRA (Ventana Medical Systems Inc., Tucson, AZ, USA). Study patients were either part of the French Network on Pulmonary Hypertension, a program approved by the institutional Ethics Committee (Protocol N8CO-08-003, ID RCB: 2008-A00485-50, approved on 18 June 2008) or were collected at the Erasmus MC Rotterdam and approved by the Medical Ethical Committee of the Erasmus MC Rotterdam (METC 2012-512). From all study patients, informed consent for collection of material was obtained. Slides of 4 µm thick formalin-fixed paraffin-embedded (FFPE) sections were stained for CD8, CD68, α-SMA and CD206. All antibodies are listed in Supplementary Table S2. In brief, following deparaffinization and heat-induced antigen retrieval with CC1 (#950-224, Ventana) for 32 min, anti-CD8 was incubated for 32 min at 37 °C followed by omnimap anti-rabbit HRP (#760-4311, Ventana) and detection with chromomap DAB (#760-159, Ventana). An antibody denaturation step was performed with CC2 (#950-123, Ventana) at 100 °C for 20 min. Secondly, incubation with anti-CD68 was performed for 16 min at 37 °C, followed by omnimap anti-mouse HRP (#760-4310, Ventana) and detection with Discovery Purple (#760-229, Ventana) for 32 min. An antibody denaturation step was performed with CC2 at 100 °C for 20 min. Thirdly, anti-α-SMA was incubated for 32 min at 37 °C, followed by omnimap anti-mouse HRP (#760-4310, Ventana) and detection with Discovery Green (#760-271, Ventana) for respectively 32 and 16 min. An antibody denaturation step was performed with CC2 at 100 °C for 20 min. Lastly, sections were incubated with anti-CD206 for 32 min at 37 °C followed by mouse-NP (#760-4816, Ventana) for 24 min at 37 °C and subsequently anti-NP-AP (#760-4827, Ventana) for 16 min and detection with Discovery Yellow (#760-239, Ventana) for 44 min. Incubation was followed by hematoxylin II counter stain for 4 min and then a blue coloring reagent for 4 min according to the manufacturer’s instructions (Ventana).

### 4.6. Flow Cytometry

To obtain single-cell suspensions, lung and heart tissues were digested using DNase and Liberase at 37 °C for 30 min. After digestion, lungs and heart were homogenized through a 100-µm cell strainer (BD Bioscience) and red blood cells were lysed using an osmotic lysis buffer. Flow cytometry extracellular and intracellular staining procedures have been described previously [44]. Dead cells were excluded using Fixable Viability Dye Live/Dead eF506 (eBioscience, San Diego, CA, USA). Monocloncal antibodies used are listed in Supplementary Table S3. Data was acquired using an LSR II flow cytometer (Beckton Dickinson, Franklin Lakes, NJ, USA) with FACS software (Beckton Dickinson). Data analysis was performed using FlowJo version 10 (Tree Star Inc software, Ashland, OR, USA).

### 4.7. Statistic Analysis

For comparisons between *Tnfaip3^DNGR1-WT^* and *Tnfaip3^DNGR1-KO^* mice, or between the TLR stimulation and NaCl stimulation, a Mann–Whitney U test was used. For comparisons between multiple groups (WT, *Tnfaip3^DNGR1-KO^, BMPR2^+/R899X^* and *Tnfaip3^DNGR1-KO^* X *BMPR2^+/R899X^* mice) a Kruskal—Wallis test was used, in combination with a Dunn’s multiple comparison test comparing either mouse group to the WT mouse group or comparing all groups. *p*-values of <0.05 were considered statistically significant. Statistical analysis was performed using Prism 8 (GraphPad Software, V8.0, San Diego, CA, USA).

## Data Availability

The data presented in this study are available in this article and in the supplementary material.

## Supplementary Materials

The supplementary materials are available online at https://www.mdpi.com/1422-0067/22/4/1756/s1.

## Abbreviations

AM	Alveolar macrophages
BATF3	Basic leucine zipper transcriptional factor ATF-like 3
BMPR2	Bone morphogenetic protein receptor type 2
cDC1s	Type 1 conventional DCs
cDC2s	Type 2 conventional DCs
CTD-PAH	Connective tissue disease PAH
CXCL8	X-X-C-motif chemokine ligand 8
DCs	Dendritic cells
EvG	Elastic von Gieson
H&E	Hematoxylin and eosin
HPAH	Heritable PAH
i.p.	Intraperitoneal
i.t.	Intratracheally
IL	Interleukin
IM	Interstitial macrophages
IPAH	Idiopathic PAH
LPS	Lipopolysaccharide
LV	Left ventricle
Mo-DC	Monocyte-derived-DC
NaCl	Sodium chloride
NF-kB	Nuclear factor-kappa B
PAH	Pulmonary arterial hypertension
PAP	Pulmonary arterial pressure
PASMCs	Pulmonary artery smooth muscle cells
PH	Pulmonary hypertension
pDC	Plasmacytoid DC
PFA	paraformaldehyde
Poly I:C	Polyinosinic-polycytidylic acif high molecular weight
RV	Right ventricle
RVSP	Right ventricular systolic pressure
S	Septum
TGFβ	Transforming growth factor-β
TLOs	Tertiary lymphoid organs
TLR	Toll like receptor
*Tnfaip3*	TNFα-induced protein-3
WHO	World health organization
WT	Wild type
α-SMA	Alpha smooth muscle actin
