# Supplementary material for: Central Role of Dendritic Cells in Pulmonary Arterial Hypertension in Human and Mice
Source: Int J Mol Sci. 2021 Feb 10;22(4):1756. doi: 10.3390/ijms22041756 (PMC7916474; doi:10.3390/ijms22041756)
Supplement: Supplementary file 1 [file ijms-22-01756-s001.pdf]

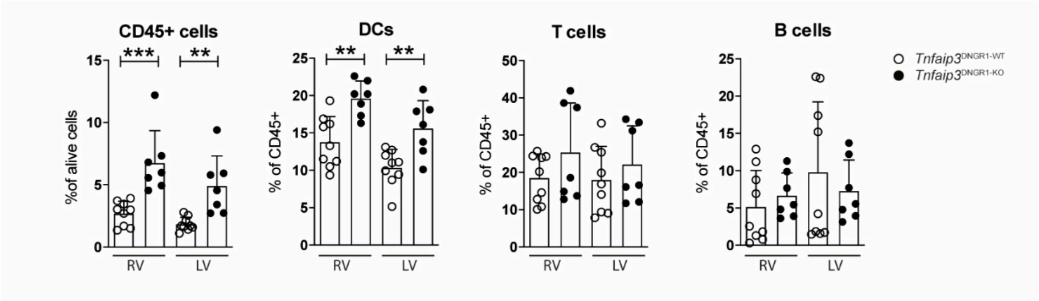

Supplementary Figure S1. Flow cytometry analysis in hearts of 31-week-old *Tnfaip3*$^{DNGR1-KO}$ mice shows increased proportions of CD45+ cells and DCs, both in right and left ventricle. Flow cytometry analysis for CD45+ cells (alive, CD45^{+}), DCs (CD3-/CD19-, MHC-II+CD11c+), T cells (CD3+) and B cells (CD19+) in separately measured right ventricle (RV) and left ventricle (LV) cell suspensions from hearts of *Tnfaip3*$^{DNGR1-WT/KO}$ mice. Results are presented as mean values + standard deviation of 7-9 mice per group. *$P < 0.05$, **$P < 0.01$, ***$P < 0.001$.

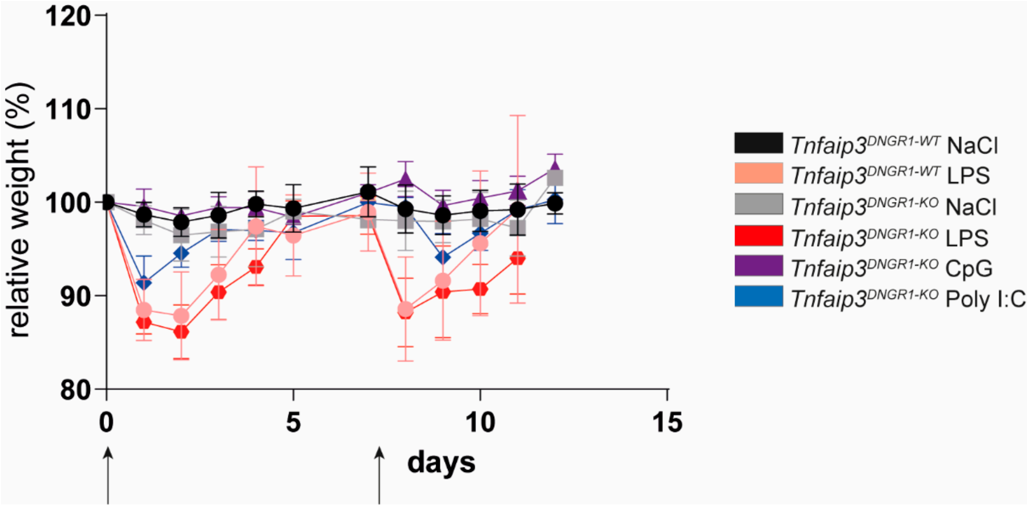

Supplementary Figure S2. Weight curve of TLR-exposed WT and *Tnfaip3*$^{DNGR1-KO}$ mice. Weight curve of WT and *Tnfaip3*$^{DNGR1-KO}$ mice, exposed to TLR4 ligand lipopolysaccharide (LPS), TLR3 ligand polyinosinic-polycytidylic acid (Poly I:C) or TLR9 ligand CpG. Arrows indicate moments of i.t. TLR exposure. For each mouse the weight at day 0 was set to 100%. Data are shown as mean values + standard deviations from 5-10 mice per group.

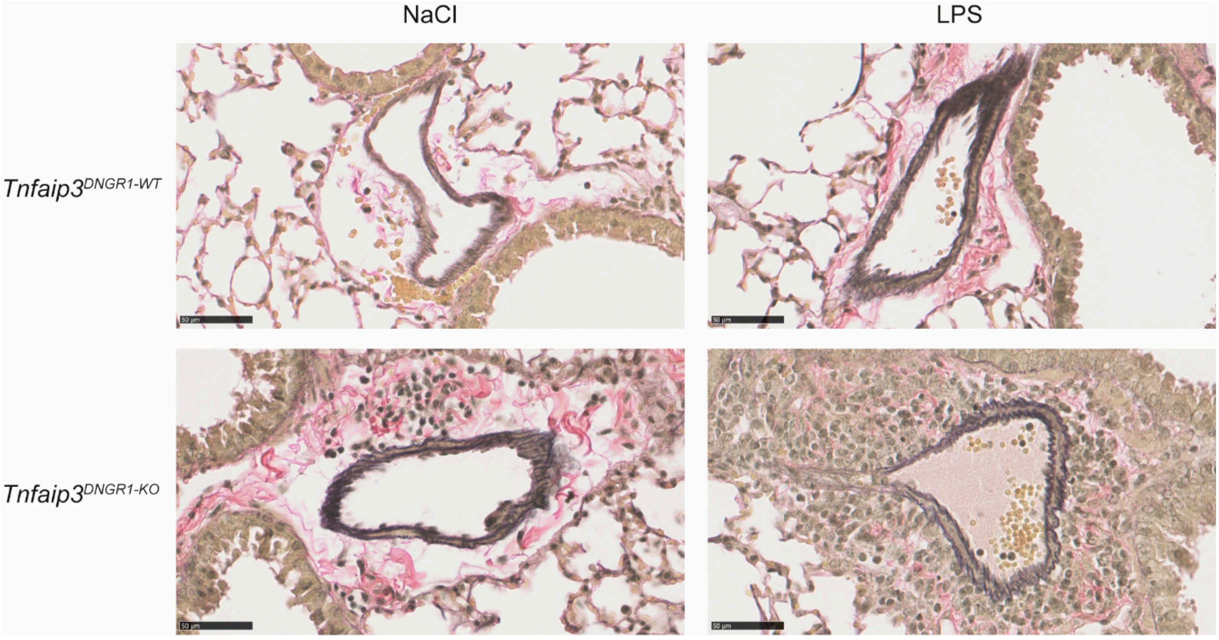

Supplementary Figure S3. LPS-exposure in WT and *Tnfaip3*$^{DNGR1\text{-}KO}$ mice leads to thickening of vessels. Elastin van Gieson (EvG) staining in lung tissue of saline- and LPS-exposed WT and *Tnfaip3*$^{DNGR1\text{-}KO}$ mice showing thickening of mid-sized vessels. Data shown are representative for 4-6 mice per group.

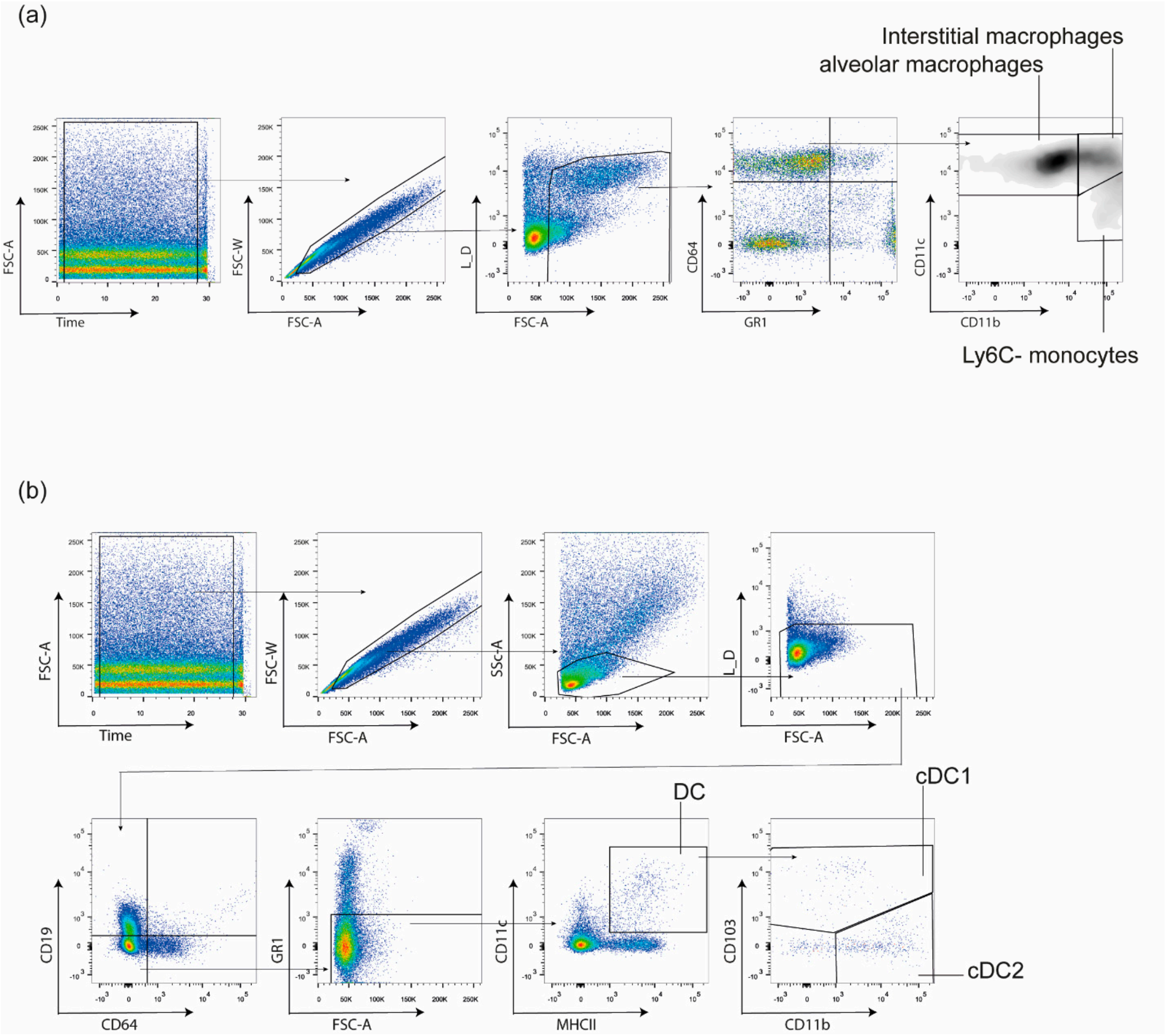

Supplementary Figure S4. Gating strategy for lung myeloid cell populations. (**a**) Flow cytometric gating of lung macrophages and Ly6C- monocytes; (**b**) flow cytometric gating strategy of cDC subsets in the lung.

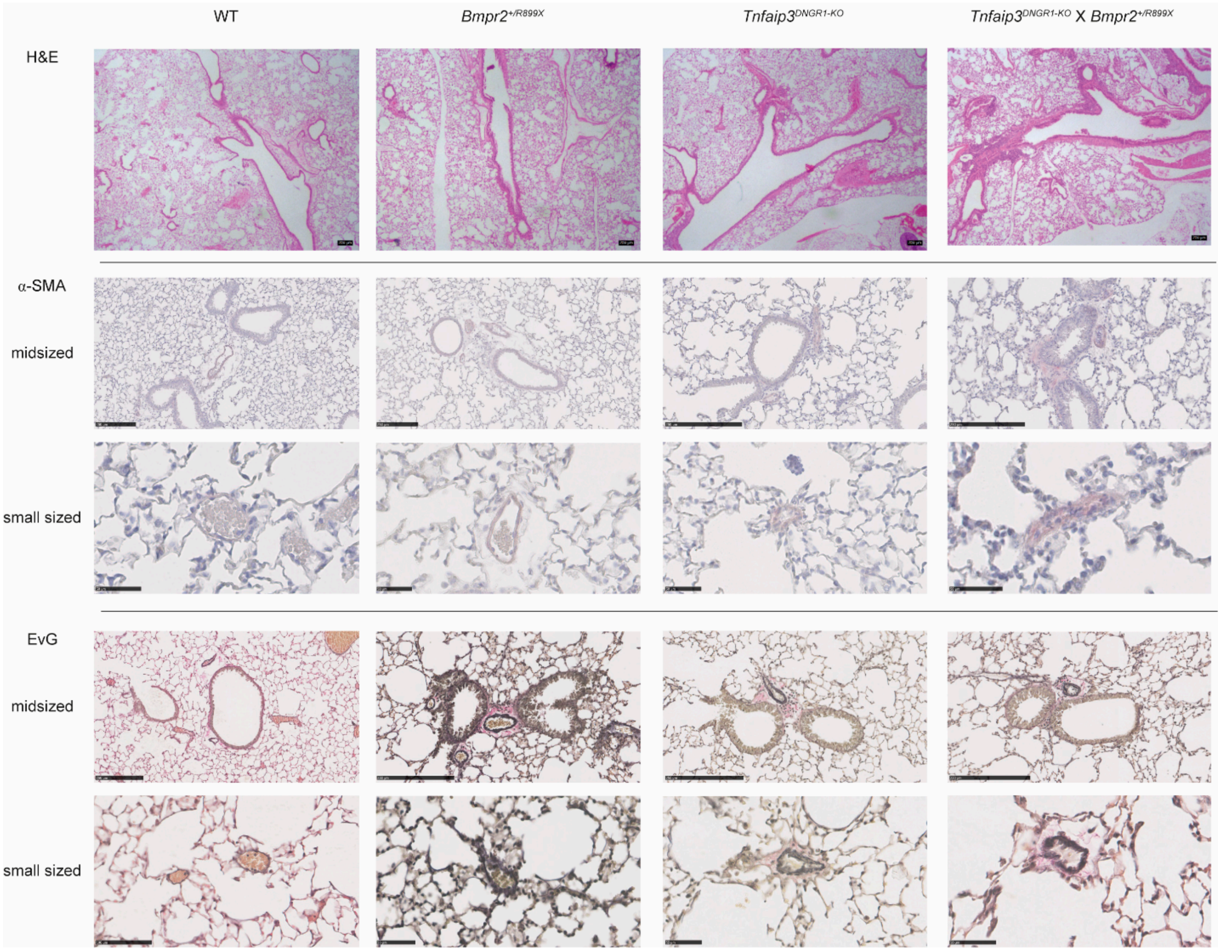

Supplementary Figure S5. Remodeling of small and midsized vessels in *Tnfaip3*$^{DNGR1\text{-}KO}$, *Bmpr2*$^{+/R899X}$ and *Tnfaip3*$^{DNGR1\text{-}KO}$ X *Bmpr2*$^{+/R899X}$. Hematoxylin and eosin (H&E), smooth muscle acting staining (α-SMA) and Elastin van Gieson (EvG) staining in lung tissue of WT, *Bmpr2*$^{+/R899X}$, *Tnfaip3*$^{DNGR1\text{-}KO}$ and *Bmpr2*$^{+/R899X}$ X *Tnfaip3*$^{DNGR1\text{-}KO}$ mice. Data shown are representative for 6-8 mice per group.

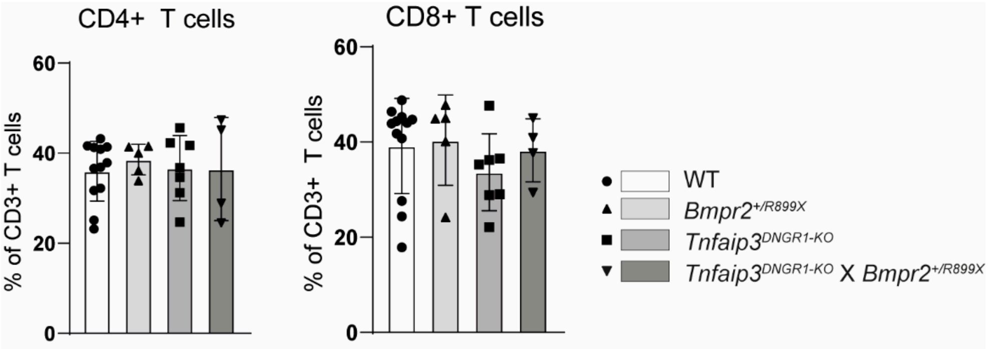

Supplementary Figure S6. Proportions of CD4+ T cells and CD8+ T cells are similar between WT, *Tnfaip3*$^{DNGR1\text{-}KO}$, *Bmpr2*$^{+/R899X}$ and *Tnfaip3*$^{DNGR1\text{-}KO}$ X *Bmpr2*$^{+/R899X}$ mice. Quantification of CD4+ T cells and CD8+ T cells (as proportions of total CD3+ T cells) in the lungs of the indicated mouse groups, determined by flow cytometry. Results are presented as mean + standard deviation of 4 – 12 mice per group.

Supplementary Table S1. RT-PCR primers.

Primer	Sequence
CD11c FW	GGCTGCAAGCATCATTCGTT
CD11c RW	AGCCTTCCCCTGGTTCCATA
BATF3 FW	CTTTGTGCAGCTTCGGTCAG
BATF3 RW	GTGTGCAAACCAAGGTTCCG
CD4 FW	CCAGCTGTCTGCTTGGATCA
CD4 RW	GCCCTCTCGTAAACTGTGCT
CD8 FW	GTTCCAGTTTCGGGGTCCAT
CD8 RW	CACCAGTCAGTGAGGGTTCC
RQ GAPDH FW	TTCACCACCATGGAGAAGGC
RQ GAPDH RW	GGCATGGACTGTGGTCATGA

Supplementary Table S2. Monoclonal antibodies used for histology.

Antibody	Type	Clone	Company
α-SMA-PE	Mouse anti-human	IA4	R&D
CD11c PE	Hamster anti-mouse	N418	ThermoFisher scientific
CD3 APC-ef780	Rat anti-mouse	17A2	ThermoFisher scientific
CD8 Fitc	Rat anti-mouse	53-6.7	BD
CD8	Rabbit anti-human	SP57	Ventana
CD68	Mouse anti-human	KP1	Ventana
CD206	Mouse anti-human	685645	R&D
α-Sma	Mouse anti-human	1A4	Ventana
MHCII-bio	Rat anti-mouse	MRC OX-6	Abcam
GFP	Rabbit anti-GFP	polyclonal	Abcam

Supplementary Table S3. Monoclonal antibodies used for flow cytometry.

Antibody	Conjugate	Clone	Company
CD40	PE	1C10	eBioscience
CD11c	PE-TXR	N418	Invitrogen
CD11b	PercCP-Cy5.5	M1/70	BD
GR1	PE-Cy7	RB6.8C5	eBioscience
CD64	AF647	X54-5/7.1	BD
MHCII	AF700	M5/114.15.3	eBioscience
CD19	APC-Cy7	1D3	BD
CD103	eF450	2E7	eBioscience
CD86	BV650	FUN1	BD
PD-L1	BV711	MIH5	BD
CD8	Fitc	53-6.7	eBioscience

CD19	PerCP-Cy5	eBio1D3	eBioscience
CD3	APC-Cy7	17A2	eBioscience
KI-67	FITC	SolA15	eBioscience
CD25	PercP-Cy5.5	PC61	BD
CD8	Pe-Cy7	53-6.7	eBioscience
CD62L	APC	MEL-14	eBioscience
FOXP3	AF700	FJK-16s	eBioscience
CD3	BV421	145-2c11	BD
CD44	BV650	IM7	BD
CD4	BV711	RM4-5	BD
MHCII	Biotine	M5/114.15.2	eBioscience
Streptavidin	BV786		BD
CD45	PerCP-Cy5.5	30-F11	eBioscience
CD19	APC	1D3	BD
